# Karyotyping of aneuploid and polyploid plants from low coverage whole-genome resequencing

**DOI:** 10.1186/s12870-023-04650-9

**Published:** 2023-12-08

**Authors:** Kanglu Zhao, Yanbo Bai, Qingyu Zhang, Zhen Zhao, Yao Cao, Lu Yang, Ni Wang, Junxiong Xu, Bo Wang, Lei Wu, Xiufeng Gong, Tuanrong Lin, Yufeng Wang, Wei Wang, Xingkui Cai, Yuhe Yin, Zhiyong Xiong

**Affiliations:** 1 Key Laboratory of Herbage and Endemic Crop Biology, Ministry of Education, School of Life Sciences, Inner Mongolia University, Hohhot, 010070 China; 2 Institute of Ulanqab Agricultural and Forestry Sciences, Inner Mongolia, Ulanqab, 012000 China; 3 Key Laboratory of Horticultural Plant Biology, Key Laboratory of Potato Biology and Biotechnology, Ministry of Agriculture and Rural Affairs, College of Horticulture and Forestry Sciences, Ministry of Education, Huazhong Agricultural University, Wuhan, 430070 China

**Keywords:** Karyotype, Aneuploid, Polyploid, Whole-genome resequencing, FISH

## Abstract

**Background:**

Karyotype, as a basic characteristic of species, provides valuable information for fundamental theoretical research and germplasm resource innovation. However, traditional karyotyping techniques, including fluorescence in situ hybridization (FISH), are challenging and low in efficiency, especially when karyotyping aneuploid and polyploid plants. The use of low coverage whole-genome resequencing (lcWGR) data for karyotyping was explored, but existing methods are complicated and require control samples.

**Results:**

In this study, a new protocol for molecular karyotype analysis was provided, which proved to be a simpler, faster, and more accurate method, requiring no control. Notably, our method not only provided the copy number of each chromosome of an individual but also an accurate evaluation of the genomic contribution from its parents. Moreover, we verified the method through FISH and published resequencing data.

**Conclusions:**

This method is of great significance for species evolution analysis, chromosome engineering, crop improvement, and breeding.

## Background

The number and morphology of chromosomes describe a karyotype, which is a fundamental characteristic of all organisms [1]. Karyotype analysis, an important technique for chromosome examination and genetic background screening, is widely used in prenatal diagnosis [2], species evolution analysis [3], chromosome engineering [4], etc. The traditional method of chromosome identification compares chromosome morphology, including length, arm ratio, and secondary constriction position [5]. However, chromosomal morphology may vary depending on material processing methods or cell cycle, with accurate results not being obtained [6]. Researchers resorted to staining the chromosomes with chemicals to identify them by their constant striation features using the chromosomal banding technique [7]. However, due to the tight superhelix structure of chromosomes in some plants, the bands were not obvious or absent after staining, making this method not applicable universally [8].

The development of the fluorescence in situ hybridization (FISH) technique marked the transition from the classical cytogenetics era to the modern molecular cytogenetics era [9]. FISH hybridizes fluorescent-labeled nucleic acid fragments (probes) to denatured genomic DNA based on the principle of complementary base pairing, and identifies chromosomes by detecting the number and arrangement of signals on them using a fluorescence microscope. The widely used techniques include (1) genome in situ hybridization (GISH), which uses whole genome sequences as probes to distinguish different chromosomal sets [10, 11], (2) Multi-color FISH, which uses the characteristics of high copies and special distribution on chromosomes of repeated sequences combined with polychromatic probes to identify chromosomes [12], (3) BAC (bacterial artificial chromosome)-FISH, where large DNA fragment cloning vectors marked as probes are used to identify chromosomes [13, 14], and (4) Oligo-FISH, where chromosome-specific oligonucleotides are first designed based on a reference genome. Oligo-FISH requires no extensive library screening, is flexible in design, and is also not limited to special regions of chromosomes (such as telomeres, centromeres, and rDNA sites). It also offers several advantages compared to traditionally prepared probes, including consistent probe quality and less time for probe preparation [15, 16]. Notably, whole-chromosome oligo-FISH paints using synthetic oligonucleotide libraries can be applied to visualization of simple or complex chromosomal aberrations, establishment of chromosomal domains, illustration of mitotic and meiosis behavior, and providing of insights into chromosomal relationships for genetically diverse lines [17].

However, the application of the FISH techniques is mainly limited by the lack of robust DNA probes in most plant species, especially non-model plants [9, 18]. Besides, a series of technical challenges were encountered in FISH, including the hardness of the plant cell wall and the density of the microsporocyte’s cytoplasm, hampering the accessibility of the probes to the chromosomes [19]. For example, the development of karyotypes for *Brassica* was challenging due to their small chromosome size and the lack of distinct karyological features in the metaphase chromosomes [13, 20]. Thus, although great progress has been achieved on FISH, there are still many disadvantages, including only a few plants having chromosome identification protocols, special tissues at special growth periods being required as materials, the experimental process being time-consuming, consumables being expensive, the testing equipment having high requirements, aneuploids and polyploids having too many chromosomes to spread out during tissue preparation, specific information about the variations cannot be provided, etc. [9, 18, 21].

Compared to FISH, molecular karyotyping has better resolution, a higher degree of automation, and a faster detection cycle. For example, chromosomal microarray analysis (CMA) involves molecular hybridization of a labeled sample with DNA probes covering important segments of the chromosome, and analysis of the hybridization signals yielding the molecular number and sequence information of the sample [22]. However, due to the limitation of microarray design, the copy number variation (CNV) of uncovered genomic regions on the platform cannot be detected. The application field of CMA is mostly prenatal diagnosis and is yet to be popularized in plants.

Plant scientists face multiple challenges, particularly those working on crop improvement and breeding. Advances in genome sequencing and resequencing play a role in meeting these challenges [23]. By the end of 2020, 1031 genomes of 788 different plant species were sequenced and published, of which 360 species have genomes assembled to the chromosome level [24]. These data provide a great choice for obtaining the copy numbers of each chromosome using whole genome resequencing to make molecular karyotypes by aligning to the genome at the chromosome level [25, 26]. Its advantages are as follows: DNA can be extracted from any material; low coverage resequencing is cheap; results can be obtained from a computer in half an hour; specific variation positions can be obtained; the analysis process is easy to repeat; and samples can be analyzed in batches.

However, published methods infer chromosomal copy numbers from standardized or normalized read counts combined with statistical tests and require a control sample [25, 26], thus presenting a significant challenge for wet-lab biologists. We assumed that the copy number of chromosomes can be inferred from large CNVs. According to Smolander et al. ‘s evaluation, BIC-seq2 and FREEC are the two best-performing tools for identifying large CNVs from low coverage whole-genome resequencing (lcWGR) data [27]. Because FREEC has a much shorter runtime (~ 3min) than BIC-seq2 (> 3 h), and contains ploidy setting parameters, it is reasonable to assume that FREEC is the most suitable tool for the CNV analysis in both aneuploid and polyploid plants. If these aneuploid or polyploid plants are newly generated hybrids, it is necessary to analyze their genome constitutes. Published pipelines such as VcfHunter (https://github.com/SouthGreenPlatform/VcfHunter) use large populations to study the evolution and domestication of crops hundreds or even millions of years ago, performing chromosome painting of accessions based on the contribution of ancestral groups [28]. These pipelines are not suitable for the analysis of a small number of newly generated hybrids. Therefore, we refer to the theory of QTL-seq [29], using variations (SNP and InDel) to characterize genome structure along chromosomes. Finally, we proposed a simpler and more accurate karyotyping pipeline for plants, which was verified through FISH and published resequencing data. Our method was used to demonstrate gene flow from diploid to allopolyploid plants using triploid plants as a bridge [30].

## Results

### Molecular karyotypes are consistent with cytogenetic karyotypes and have a great advantage

**Fig. 1:**
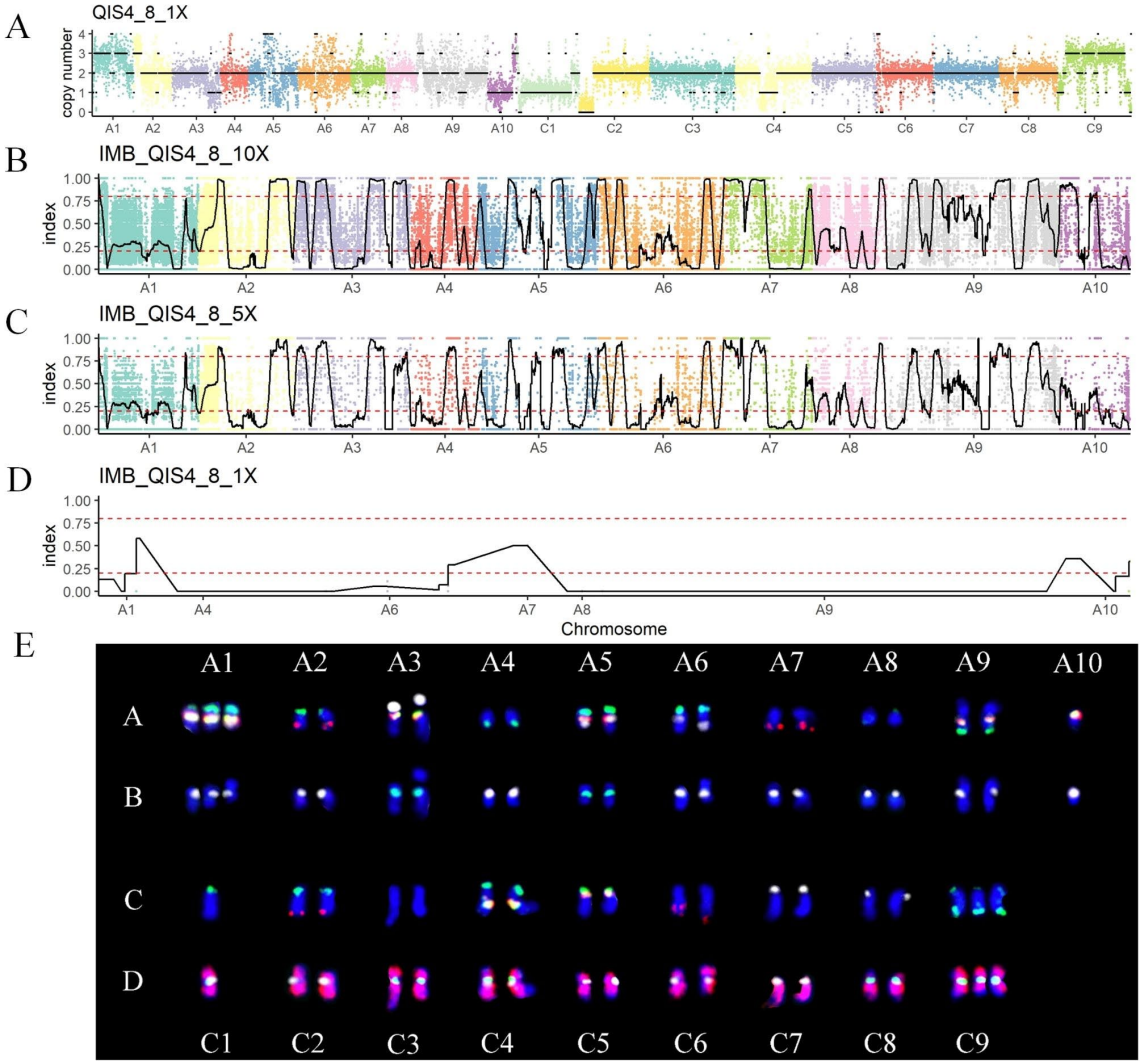
Karyotyping of the resynthesized *B. napus* QIS4_8. (A) Molecular karyotype of the QIS4_8 with 1× sequence depth. The scatter represents Ratio * 2 (expected ploidy), and the black line shows the copy number of chromosomes. (B, C, and D) Genotyping of the QIS4_8 with different sequence depths (10, 5, and 1×). The scatter represents the index of each position. The black line was obtained by sliding window analysis. The two red dotted lines divide the average index (black line) into three regions as previously described [30]. Thus, 0-0.2 (variation type from Quinta) and 0.8-1 (IMB218 alleles) are homozygous regions, and 0.2–0.8 is the heterozygous region. (E) For cytogenetic karyotype analysis, the A chromosomes are shown in lanes A and B, and the C chromosomes are shown in lanes C and D. The first round of FISH included 45 S rDNA (white), 5 S rDNA (yellow), BAC clone KBrB072L17 (green), and KBrH092N24 (red) probes, and the hybridization results are shown in lanes A and C. The second round of FISH included CentBr1 (white), CentBr2 (green), and BAC BNIH 123L05 (red) probes containing C genome-specific repeated sequences, and the hybridization results are shown in lanes B and D. One near-tetraploid QIS4_8 with 38 chromosomes derived from the A_n_A_r_C_n_ allotriploid

**Fig. 2:**
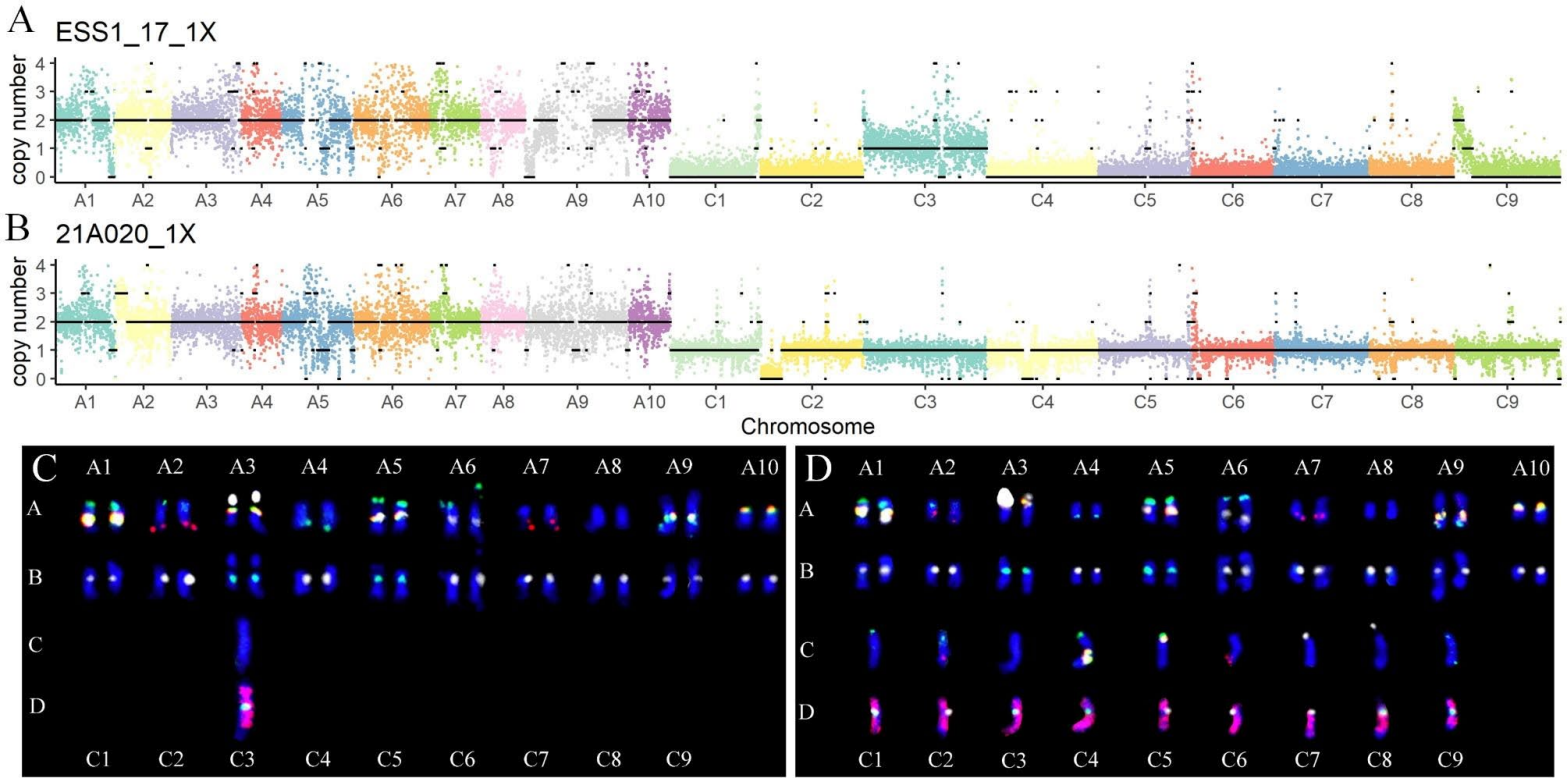
Karyotyping for different ploidy rapeseeds. (A) For molecular karyotypes of ESS1_17 with 1× sequence depth, the scatter represents Ratio * 2 (expected ploidy). (B) For molecular karyotypes of 21A020 with 1× sequence depth, the scatter represents Ratio * expected ploidy (2 for subgenome A; 1 for subgenome C). The black line shows the copy number of chromosomes. (C-D) For cytogenetic karyotype analysis, the A chromosomes are shown in lanes A and B, and the C chromosomes are shown in lanes C and D. The first round of FISH included 45 S rDNA (white), 5 S rDNA (yellow), BAC clone KBrB072L17 (green), and KBrH092N24 (red) probes, and the hybridization results are shown in lanes A and C. The second round of FISH included CentBr1 (white), CentBr2 (green), and BAC BNIH 123L05 (red) probes containing C genome-specific repeated sequences, and the hybridization results are shown in lanes B and D. (C) A MAAL ESS1_17 with two sets of A genomes plus one more C3 chromosome. (D) An allotriploid 21A020 is composed of two sets of A genomes and one set of C genome

**Fig. 3:**
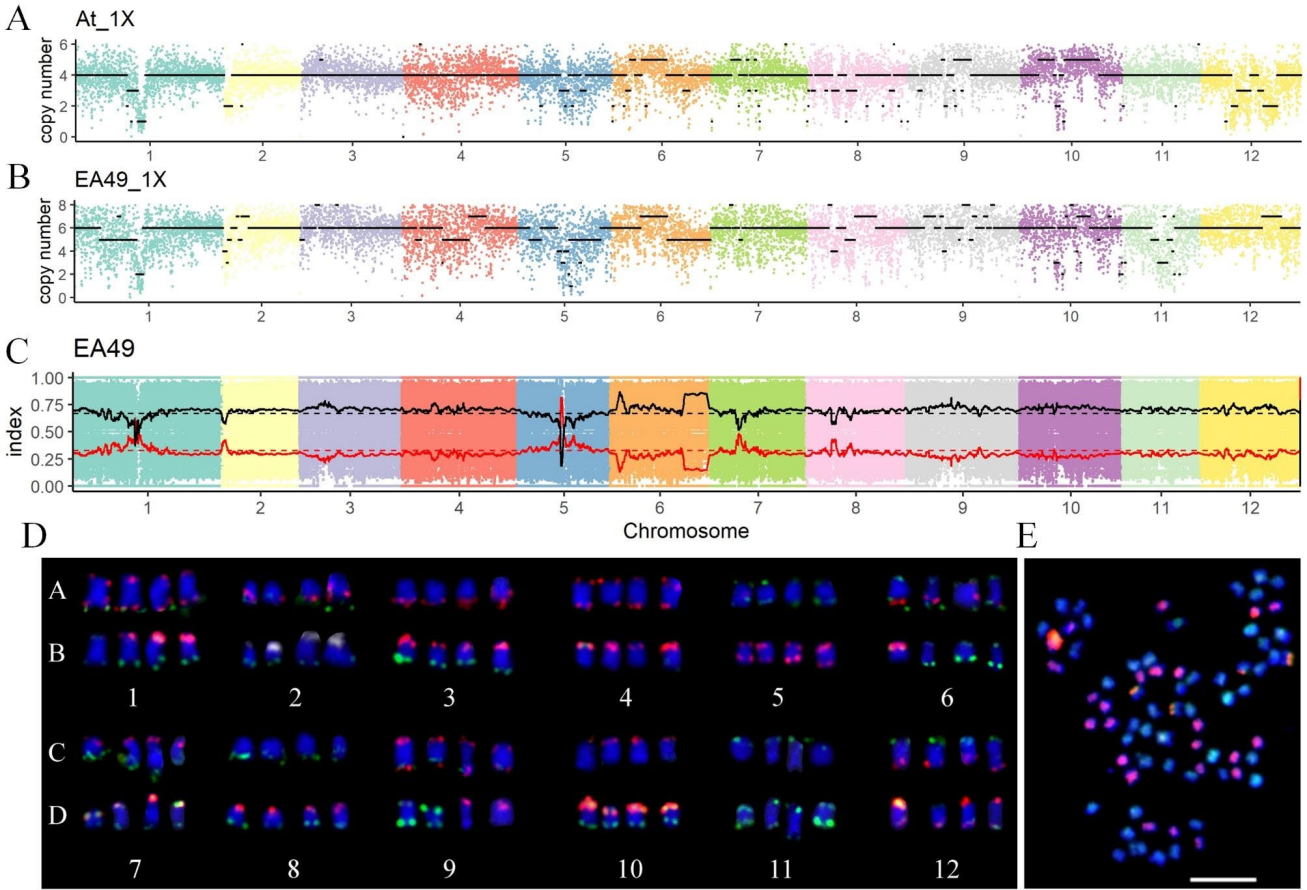
Karyotyping for different ploidy potatoes. Molecular karyotypes of At (A), and EA49 (B) with 1× sequence depth. The scatter represents Ratio * expected ploidy (4 for At; 6 for EA49), and the black line shows the copy number of chromosomes. (C) Genotyping of the EA49 with 10× sequence depth. Using *S. etubersoum* (EE) as the reference parent to calculate the index, and the scatter represents the index of each position. The black line, obtained by sliding window analysis, represents the proportion (2/3, black dotted line) of AC142 (AA) genotypes. The red line is “1 - black line”, representing the ratio (1/3, red dotted line) of EE genotypes. (D) FISH mapping of At chromosomes using two oligo-FISH probes (lanes A and C). The same cell was reprobed with subtelomeric repeated sequences CL34 (green), CL14 (red), and 45 S rDNA (white) probes (lanes B and D). At is an autotetraploid with 48 chromosomes. (E) GISH mapping of EA49 chromosomes using two genomic probes (AA, green; EE, red). EA49 is a hexaploid with 72 chromosomes consisting of two sets of A genomes and one set of E genome. Scale bar, 10 μm

According to the method described, molecular and cytogenetic karyotypes of allopolyploid rapeseeds and autopolyploid potatoes were analyzed simultaneously. Molecular karyotypes were analyzed using the resequencing data with 1× depth. The karyotype of QIS4_8 showed that it was a recessive aneuploid (2n = 38, euploidy alike) with two pairs of homoeologous chromosomes dosage variations (three A1-one C1; one A10-three C9) and two copies of the other chromosomes (Fig. 1A). ESS1_17 was a monosomic alien addition line (MAAL, AA + C3) composed of two sets of A genomes and one C3 chromosome (Fig. 2A). The 21A020 was an allotriploid with two sets of A genomes and one set of C genome (Fig. 2B). Moreover, molecular karyotypes were also applied to autopolyploids, including tetraploid At (Fig. 3A) and hexaploid EA49 (Fig. 3B), having four and six sets of genomes, respectively.

For cytogenetic karyotypes, combining two rounds of hybridization and seven probes (four in the first round and three in the second round), every chromosome from the A and C genomes of *Brassica* was unambiguously identified. QIS4_8 was found to contain 38 chromosomes with three A1 and one homoeologous C1 chromosomes, and one A10 and three homoeologous C9 chromosomes (Fig. 1E). ESS1_17 was a MAAL, having two sets of A genomes plus one more C3 chromosome (Fig. 2C), while 21A020 was an allotriploid composed of two sets of A genomes and one set of C genome (Fig. 2D). Two FISH techniques, namely Oligo-FISH and Multi-color FISH using two and three probes, respectively, were then used to identify the potato karyotypes. The result showed that At was an autotetraploid (Fig. 3D).

Finally, molecular and cytogenetic karyotypes were compared, and it was found that the karyotypes obtained by both methods were highly consistent (Figs. 1A and E, 2A, C, B and D and 3A and D). Despite technicians using the cytogenetic technique for seven years, misidentification of C1 and C5 chromosomes of *Brassica* still occurred due to their similar signals (especially hybridization is less effective) and chromosome length. However, this problem was absent in molecular karyotyping. Notably, molecular karyotyping not only identified each chromosome and obtained the copy number, but also showed the loss and duplication of partial chromosomal segments. For example, QIS4_8 had three A1 chromosomes and one homoeologous C1 chromosome (Fig. 1A, E), with the ends of both A1 (about 32–38 Mb) and C1 (about 50–58 Mb) chromosomes having two copies, as a result of homoeologous exchange [31].

### The molecular karyotyping pipeline in this study can reproduce the results of published molecular karyotypes

**Fig. 4:**
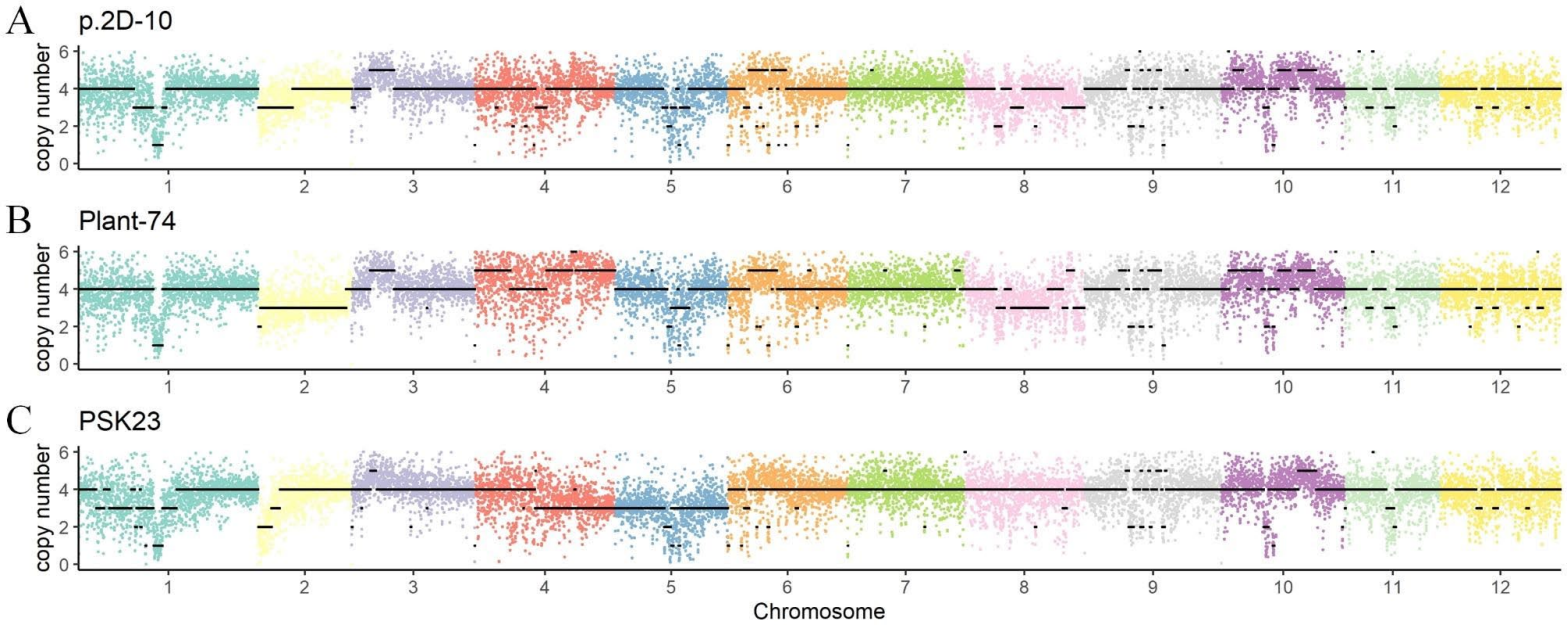
Karyotyping for potato published data. Molecular karyotypes of p.2D-10 (A), Plant-74 (B), and PSK23 (C). The scatter represents Ratio * 4 (expected ploidy), and the black line shows the copy number of chromosomes

Further, published resequencing data of *Solanum tuberosum* cv Desiree for karyotyping [25] was used to verify our method in this study. Consistent with previous studies, p.2D-10 was found to be an autotetraploid (Fig. 4A), while Plant-74 (Fig. 4B) and PSK23 (Fig. 4C) were found to be aneuploid. Plant-74 lost one chromosome 2 and part of chromosome 8, gaining an extra chromosome 4. PSK23 lost one chromosome 5 and half of chromosome 4. Thus, molecular karyotypes derived from different methods showed similar genome dosages, including plants propagated by stem cutting (Fig. 4A), and plants regenerated from protoplasts (Fig. 4B) and stem internodes by *Agrobacterium*-mediated transformation (Fig. 4C). Some samples were sequenced at depths as low as 0.4×, with accurate karyotypes being obtained. In conclusion, if only chromosome copy number is analyzed, this study suggested sequencing 1× for small genome species or small sample size, while the sequencing depth for large genome species or large sample size can be reduced appropriately (at least 0.01x [27]). As for the appropriate minimum depth for target species, researchers can use the software seqtk to extract different depths for testing.

### Inference of homologous chromosome and genome origin based on molecular karyotypes

In addition to chromosome copy numbers, genotypes of the offspring of hybrids can be inferred if their parental resequencing data is available. For example, QIS4_8 is an offspring of a triploid hybrid (2n = 29, A_n_A_r_C_n_) crossed between allotetraploid *B. napus* Quinta and diploid *B. rapa* IMB218, and its C genome is derived from *B. napus*, while the A genome has different origins. Therefore, after aligning samples to the rapeseed reference genome (containing A and C chromosomes), only the A genome of QIS4_8 was analyzed. QIS4_8 was observed to have two copies of chromosome A7 (Fig. 1A, E), with the first half of the chromosome being almost the IMB218 genotype (A_r_A_r_) and the last half almost the Quinta genotype (A_n_A_n_; Fig. 1B). Besides, there were three copies of chromosome A1 (Fig. 1A, E), which most of the chromosomal fragments were observed in the heterozygous region with an average index of about 1/3, indicating that the genotype was A_n_A_n_A_r_ (Fig. 1B). Using the methods in this study, newly formed allotetraploids were successfully analyzed among the progeny of allotriploids (interploidy hybrids between *B. napus* and *B. rapa*). It was found that large chromosomal fragments and even main chromosomes came from the diploid parent *B. rapa*. The results demonstrated that genome sequences from the diploid *B. rapa* were transferred to the newly formed allotetraploids [30].

As molecular karyotypes show the origin of homologous chromosomes, different genomes can also be identified using our method. EA49 is a resynthesized hexaploid derived from a diploid potato (AA, 2n = 24) and *S. etubersoum* (EE, 2n = 24). Our pipeline analysis showed that each chromosome of EA49 has six copies (Fig. 3B) with a genotype ratio (A:E) of 2:1 (Fig. 3C), indicating that EA49 consisted of two sets of AA genomes and one set of EE genome. Further analysis with GISH demonstrated 48 A chromosomes and 24 E chromosomes (Fig. 3E). Thus, molecular karyotypes can not only replace GISH to identify different genomes but also allow the estimation of the size of the added alien chromosomal segment as well as the missing chromosomal segment described above.

If both chromosome copy number and genome structure are obtained, higher resequencing depth is required. In theory, the deeper the resequencing depth is, the more accurate the results, with the cost being higher. Here, 10× depth was found to be sufficient to obtain accurate genotyping results (Figs. 1B and 3C). Some chromosomal regions, such as A10, showed errors at 5× depth (Fig. 1C). Also, it was completely impossible to obtain valid genotypes at 1× depth (Fig. 1D) due to the minimum depth for variation detection being 3–4× [32, 33]. Therefore, it was recommended to choose the resequencing depth between 5–10×.

## Discussion

Karyotype fundamentally determines the traits of species. Accurate and rapid karyotype analysis greatly shortens the cycle of chromosome engineering [4]. Compared to euploidy, karyotype identification of aneuploidy is more challenging, especially for samples with euploidy chromosomal variations, such as QIS4_8 (2n = 38). Also, all individual chromosomes of aneuploids cannot be identified by flow cytometry and chromosome counts. Though cytogenetic karyotyping can identify each chromosome, it is challenging and low in efficiency, and aneuploid and polyploid plants have too many chromosomes to spread out during tissue preparation. However, it is easy for molecular karyotyping to identify various aneuploids and polyploids.

Compared to using standardized or normalized read counts to infer chromosome copy numbers, the pipeline in this study utilizes CNVs to make inference more convenient and accurate, without the need for control samples (optional). Control samples are not available most of the time, and in most cases, control samples with large chromosomal copy variations are not completely euploid, which can lead to analysis errors if a relative chromosomal copy is not desired. For example, Fossi et al. used p.2D-10 as a control to obtain the karyotype of Plant-74. However, chromosome 10 of p.2D-10 was not completely four copies (Fig. 4A), and so the variation of chromosome 10 in Plant-74 (Fig. 4B) was not detected [25]. This phenomenon was also observed in other chromosomes, including chromosomes 3, 5, and 6 (Fig. 4A, B). Notably, it is difficult to infer the karyotype when CNVs of a sample are too irregular, as partial CNVs cannot be reflected in chromosome copy numbers. This could also be the reason for the copy number of certain chromosomal fragments being inconsistent between the molecular and cytogenetic karyotypes.

Introgression of alien chromosomal segments containing useful genes into crop plants through wide hybridization is a valuable method for plant breeding. For example, the short arm of rye chromosome lR, carrying several disease-resistance genes, was incorporated into many high-yielding wheat cultivars [34]. Molecular karyotyping used in this study allowed the monitoring of alien chromatin during introgression. Besides, if two genomes are very closely related and share most of the repetitive DNA sequences, the distinction between the two genomes becomes relatively difficult by GISH [18]. On the contrary, molecular karyotyping helps in the most efficient and accurate identification of different genomes. The molecular karyotype system will allow karyotyping of a large number of accessions or ecotypes within species to study genetic adaptation and evolution on a chromosome scale. In addition to plants, the method can be used to analyze data for any organism [35].

## Conclusions

This study proposed a new molecular karyotyping method based on low coverage whole-genome resequencing, which had the advantages of wide application, simple operation, easy repetition, less time and cost. The method is of great significance for species evolution analysis, chromosome engineering, crop improvement, and breeding.

## Methods

### Plant materials

The resynthesized *Brassica napus* allopolyploid line EL500 (CCAA, 2n = 38) was obtained from a previous study [12]. ESS1_17 was a backcross progeny between a male parent and triploid developed by hybridizing EL500 (egg donor) with the inbred *B. rapa* parent line Si (AA, 2n = 20, pollen donor). 21A020 was a triploid developed by hybridizing the cultivar *B. napus* Quinta (AACC, 2n = 38, egg donor) with the doubled haploid *B. rapa* line IMB218 (AA, 2n = 20, pollen donor) as described previously [36]. QIS4_8 was a self-crossing progeny of the triploid 21A020. Plants were grown at 23 °C during the day and 20 °C at night with 16-h light in a growth chamber.

The somatic cell hybrid EA49 was derived from a protoplast electrofusion of the diploid potato cultivar AC142 (AA, 2n = 24) with *S. etubersoum* (EE, 2n = 24). EA49 and tetraploid cultivar At (*S. tuberosum* Atlantic, 2n = 48) were propagated by cutting nodes with an axillary bud and incubated in vitro under fixed conditions (16-h light/8-h dark, 24 °C, 40 µmol m-2s-1). To produce flower buds, plantlets from each line were shifted from tissue culture to greenhouse pots.

### Whole genome resequencing

Genomic DNA was extracted from young leaflets using the DNeasy Plant Mini Kit (Qiagen). Libraries were constructed using the KAPA Hyper Prep kit (KAPA Biosystems KK8504) throughout the protocol. Libraries were sequenced with paired-end 150 bp reads on an Illumina NovaSeq 6000 platform (Novogene, Beijing, China). Adaptor sequences and low-quality reads were trimmed by fastp software with default settings [37], and the remaining ones were called clean reads. Sequencing data of IMB218 was obtained from a previous study [38]. Sequencing data with different depths (1, 5, and 10×) were extracted from original FASTQ files using seqtk software (https://github.com/lh3/seqtk).

### Molecular karyotyping using resequencing data

**Fig. 5:**
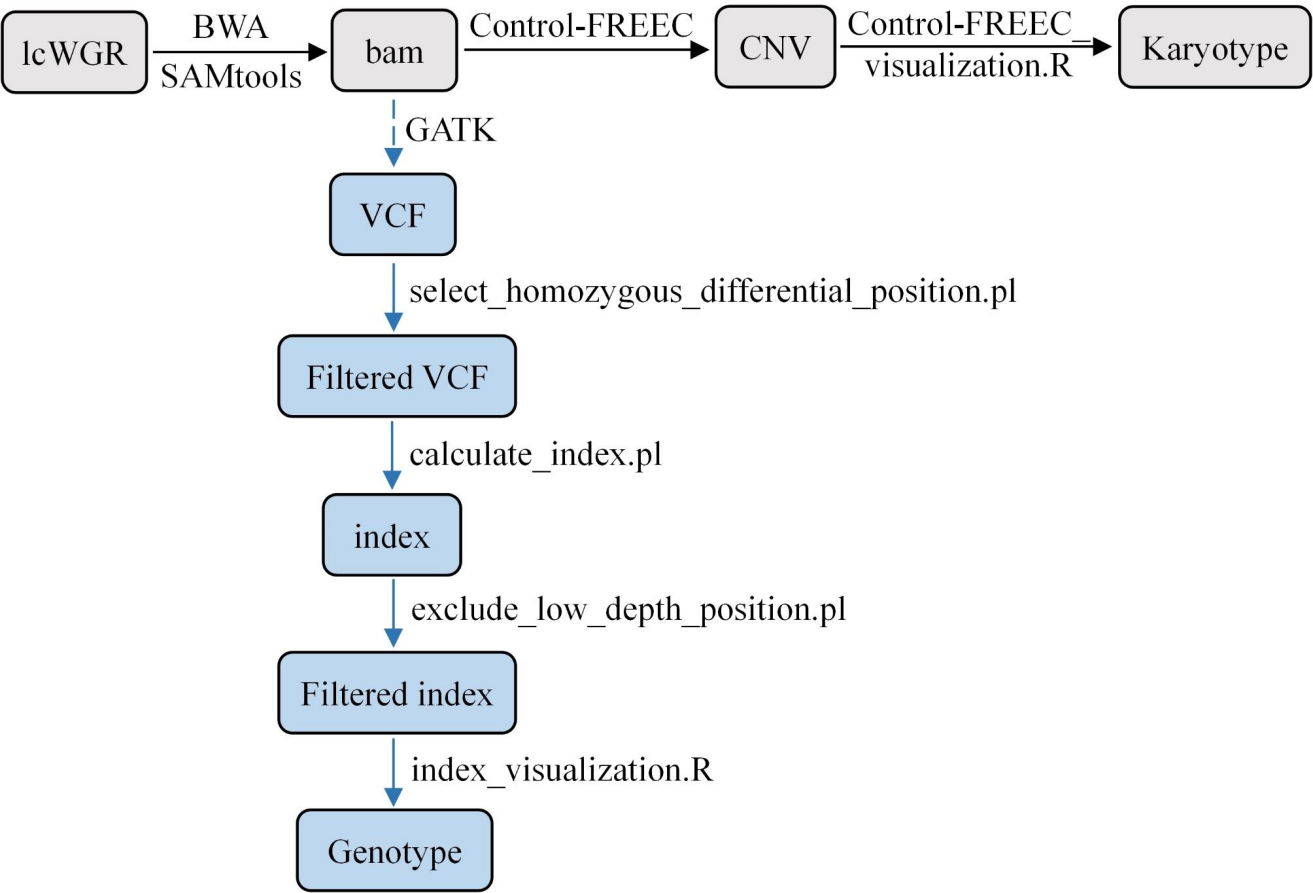
Flow chart of molecular karyotype analysis in this study. The gray background represents the chromosome copy number analysis pipeline, which requires no more than 1x depth data. The blue background represents the hybrid genome structure analysis pipeline, which requires 5-10x depth data

The flow chart of karyotype analysis is shown in Fig. 5. A computer cluster node with 48 Intel(R) Xeon(R) CPU E5-2650 at 2.20 GHz cores and 256 GB of random-access memory (RAM) was used to perform the analyses in this study. Clean reads were aligned to the reference genome of rapeseed (ZS11, contains A and C chromosomes) [39] or potato (DM v6.1) [40] using BWA software [41]. Alignment files were converted to BAM files using SAMtools [42], and applied to the absolute copy number variation (CNV) analysis by Control-FREEC [35] with default settings, except that ExpectedGC was 0.3–0.5 for rapeseeds or 0.25–0.45 for potatoes. If the subgenomic ploidy of allopolyploids, such as triploid 21A020 (AAC), is different, it is better to analyze the chromosome copy number of each subgenome separately by setting “chrLenFile” and “ploidy” parameters. Only chromosomes in the chrLenFile list will be considered by Control-FREEC, so we can analyze the A and C subgenomes separately based on the same BAM file that aligns the reference genome ZS11. If the ploidy of the material is in doubt, different values can be set and Control-FREEC will select the one that explains most observed CNVs. See the manual for detailed instructions on this tool (http://boevalab.inf.ethz.ch/FREEC/index.html). Karyotypes were then inferred from CNV visualizations by ggplot2 in R-3.6.3 (Control-FREEC_visualization.R on GitHub). All codes (including detailed notes) and test data are available on GitHub (https://github.com/kangluzhao/karyotyping).

Further, each homologous chromosome or chromosomal segment of progenies provided by which parent was distinguished and traced. The theory was similar to QTL-seq [29]. Taking QIS4_8, a progeny of Quinta and IMB218, as an example, variations (SNP and InDel) were called from BAM files with marked duplicates, and positions with QD < 2.0 or FS > 60.0 were filtered using GATK4 [43]. According to the VCF file, selecting the homozygous and differential genotype positions of parents (select_homozygous_differential_position.pl on GitHub), and then the “index” of QIS4_8 was calculated for these positions (calculate_index.pl on GitHub). If the position’s genotype of QIS4_8 exactly matches the reference parent (Quinta), we assign an index of 0. Otherwise, it’s 1 (i.e., exactly matching the IMB218). Thus, the genetic proportion of each parent could be calculated. Besides, positions with read depth < 7 were excluded (exclude_low_depth_position.pl on GitHub), as their corresponding indexes were less accurate. Finally, sliding window analysis was applied to index plots with a 2 Mb window size and 10 kb increment. The average index of the positions located in the window was calculated to reduce noise. Figures were plotted using ggplot2 in R-3.6.3 (index_visualization.R on GitHub).

### Cytogenetic karyotyping by FISH

FISH was performed on pollen mother cells at mitosis metaphase. Probes used for FISH, tissue preparation, hybridization, karyotyping, and imaging were described previously [12, 13, 44, 45].

## Data Availability

The DNA sequence is available from the National Centre for Biotechnology Information as BioProject ID PRJNA998241.

## Abbreviations

lcWGR: Low coverage whole-genome resequencing
FISH: Fluorescence in situ hybridization
GISH: Genome in situ hybridization
BAC: Bacterial artificial chromosome
MAAL: Monosomic alien addition line
CNV: Copy number variation

## References

[1] Yoshida K, Kitano J. Tempo and mode in karyotype evolution revealed by a probabilistic model incorporating both chromosome number and morphology. PLoS Genet 2021, 17(4). 10.1371/journal.pgen.1009502 PMC8081341 33861748

[2] Fan Z, Weng X, Pan Z, Fan Q, Long J, Lin G, Yang Q, Sun L (2021). Molecular diagnosis of sex chromosome mosaics in fetal amniotic cells. Med (Baltim).

[3] Moraes AP, Olmos Simoes A, Ojeda Alayon DI, de Barros F, Forni-Martins ER (2016). Detecting mechanisms of Karyotype Evolution in Heterotaxis (Orchidaceae). PLoS ONE.

[4] Zeng D, Guan J, Luo J, Zhao L, Li Y, Chen W, Zhang L, Ning S, Yuan Z, Li A (2020). A transcriptomic view of the ability of nascent hexaploid wheat to tolerate aneuploidy. BMC Plant Biol.

[5] Cheng Z, Buell CR, Wing RA, Gu M, Jiang J (2001). Toward a cytological characterization of the rice genome. Genome Res.

[6] Chen Q, Friebe B, Conner RL, Laroche A, Thomas JB, Gill BS (1998). Molecular cytogenetic characterization of Thinopyrum intermedium-derived wheat germplasm specifying resistance to wheat streak mosaic virus. Theor Appl Genet.

[7] Gill BS, Kimber G. The Giemsa C-banded karyotype of rye. *Proceedings of the National Academy of Sciences* 1974, 71(4):1247–1249. 10.1073/pnas.71.4.1247 PMC388202 4133848

[8] Anderson LK, Stack SM, Mitchell J (1982). An investigation of the basis of a current hypothesis for the lack of G-banding in plant chromosomes. Exp Cell Res.

[9] Jiang JM, Gill BS (2006). Current status and the future of fluorescence in situ hybridization (FISH) in plant genome research. Genome.

[10] Yang Y, Yan G, Li ZS, Yuan JC, Wei XC, Wei F, Tian BM, Xie ZQ, Shi GY, Zhang XW (2020). Cytological atlas at meiosis reveals insights into pollen fertility in synthetic Brassica allotriploids between allotetraploid B. carinata and diploid B. rapa. Plant Physiol Biochem.

[11] Durnam DM, Gelinas RE, Myerson D (1985). Detection of species specific chromosomes in somatic cell hybrids. Somat Cell Mol Genet.

[12] Xiong ZY, Gaeta RT, Pires JC (2011). Homoeologous shuffling and chromosome compensation maintain genome balance in resynthesized allopolyploid Brassica napus. Proc Natl Acad Sci USA.

[13] Xiong ZY, Pires JC (2011). Karyotype and identification of all homoeologous chromosomes of Allopolyploid Brassica napus and its diploid progenitors. Genetics.

[14] Jiang J, Gill BS, Wang GL, Ronald PC, Ward DC (1995). Metaphase and interphase fluorescence in situ hybridization mapping of the rice genome with bacterial artificial chromosomes. Proc Natl Acad Sci U S A.

[15] Zhang T, Liu GQ, Zhao HN, Braz GT, Jiang JM. Chorus2: design of genome-scale oligonucleotide-based probes for fluorescence in situ hybridization. Plant Biotechnol J 2021. 10.1111/pbi.13610 PMC8486243 33960617

[16] Han Y, Zhang T, Thammapichai P, Weng Y, Jiang J (2015). Chromosome-specific painting in Cucumis species using bulked oligonucleotides. Genetics.

[17] Albert PS, Zhang T, Semrau K, Rouillard JM, Kao YH, Wang CR, Danilova TV, Jiang J, Birchler JA (2019). Whole-chromosome paints in maize reveal rearrangements, nuclear domains, and chromosomal relationships. Proc Natl Acad Sci U S A.

[18] Jiang J, Gill BS (1994). Nonisotopic in situ hybridization and plant genome mapping: the first 10 years. Genome.

[19] Jeridi M, Bakry F, Escoute J, Fondi E, Carreel F, Ferchichi A, D’Hont A, Rodier-Goud M (2011). Homoeologous chromosome pairing between the A and B genomes of Musa spp. revealed by genomic in situ hybridization. Ann Botany.

[20] Snowdon RJ (2007). Cytogenetics and genome analysis in Brassica crops. Chromosome Res.

[21] Cui CH, Shu W, Li PN. Fluorescence in situ hybridization: cell-based genetic Diagnostic and Research Applications. Front Cell Dev Biol 2016, 4. 10.3389/fcell.2016.00089 PMC5011256 27656642

[22] Wapner RJ, Martin CL, Levy B, Ballif BC, Eng CM, Zachary JM, Savage M, Platt LD, Saltzman D, Grobman WA (2012). Chromosomal microarray versus karyotyping for prenatal diagnosis. N Engl J Med.

[23] Jackson SA, Iwata A, Lee SH, Schmutz J, Shoemaker R (2011). Sequencing crop genomes: approaches and applications. New Phytol.

[24] Sun Y, Shang L, Zhu Q-H, Fan L, Guo L. Twenty years of plant genome sequencing: achievements and challenges. Trends Plant Sci 2021. 10.1016/j.tplants.2021.10.006 34782248

[25] Fossi M, Amundson K, Kuppu S, Britt A, Comai L (2019). Regeneration of Solanum tuberosum plants from Protoplasts induces widespread genome instability. Plant Physiol.

[26] Wu Y, Sun Y, Sun S, Li G, Wang J, Wang B, Lin X, Huang M, Gong Z, Sanguinet KA (2018). Aneuploidization under segmental allotetraploidy in rice and its phenotypic manifestation. Theor Appl Genet.

[27] Smolander J, Khan S, Singaravelu K, Kauko L, Lund RJ, Laiho A, Elo LL. Evaluation of tools for identifying large copy number variations from ultra-low-coverage whole-genome sequencing data. BMC Genomics 2021, 22(1). 10.1186/s12864-021-07686-z PMC8130438 34000988

[28] Martin G, Cardi C, Sarah G, Ricci S, Jenny C, Fondi E, Perrier X, Glaszmann JC, D’Hont A, Yahiaoui N (2020). Genome ancestry mosaics reveal multiple and cryptic contributors to cultivated banana. Plant J.

[29] Takagi H, Abe A, Yoshida K, Kosugi S, Natsume S, Mitsuoka C, Uemura A, Utsushi H, Tamiru M, Takuno S (2013). QTL-seq: rapid mapping of quantitative trait loci in rice by whole genome resequencing of DNA from two bulked populations. Plant J.

[30] Cao Y, Zhao K, Xu J, Wu L, Hao F, Sun M, Dong J, Chao G, Zhang H, Gong X (2023). Genome balance and dosage effect drive allopolyploid formation in Brassica. Proc Natl Acad Sci U S A.

[31] Wu Y, Lin F, Zhou Y, Wang J, Sun S, Wang B, Zhang Z, Li G, Lin X, Wang X. Genomic mosaicism due to homoeologous exchange generates extensive phenotypic diversity in nascent allopolyploids. Natl Sci Rev 2020. 10.1093/nsr/nwaa277 PMC8288387 34691642

[32] Chancerel E, Lepoittevin C, Le Provost G, Lin YC, Jaramillo-Correa JP, Eckert AJ, Wegrzyn JL, Zelenika D, Boland A, Frigerio JM et al. Development and implementation of a highly-multiplexed SNP array for genetic mapping in maritime pine and comparative mapping with loblolly pine. BMC Genomics 2011, 12. 10.1186/1471-2164-12-368 PMC3146957 21767361

[33] Mamidi S, Healey A, Huang P, Grimwood J, Jenkins J, Barry K, Sreedasyam A, Shu SQ, Lovell JT, Feldman M (2020). A genome resource for green millet Setaria viridis enables discovery of agronomically valuable loci. Nat Biotechnol.

[34] Rajaram S. Adaptation, stability and high yield potential of certain 1B/1R CIMMYT wheats. In: *Proc 6th Int Wheat Genet Symp, 1983: 1983*. Maruzen: 613–621.

[35] Boeva V, Popova T, Bleakley K, Chiche P, Cappo J, Schleiermacher G, Janoueix-Lerosey I, Delattre O, Barillot E (2012). Control-FREEC: a tool for assessing copy number and allelic content using next-generation sequencing data. Bioinformatics.

[36] Gaeta RT, Pires JC, Iniguez-Luy F, Leon E, Osborn TC (2007). Genomic changes in resynthesized Brassica napus and their effect on gene expression and phenotype. Plant Cell.

[37] Chen S, Zhou Y, Chen Y, Gu J (2018). Fastp: an ultra-fast all-in-one FASTQ preprocessor. Bioinformatics.

[38] Bird KA, Niederhuth CE, Ou S, Gehan M, Pires JC, Xiong Z, VanBuren R, Edger PP. Replaying the evolutionary tape to investigate subgenome dominance in allopolyploid Brassica napus. New Phytol 2020. 10.1111/nph.17137 PMC7986222 33280122

[39] Song JM, Guan Z, Hu J, Guo C, Yang Z, Wang S, Liu D, Wang B, Lu S, Zhou R (2020). Eight high-quality genomes reveal pan-genome architecture and ecotype differentiation of Brassica napus. Nat Plants.

[40] Pham GM, Hamilton JP, Wood JC, Burke JT, Zhao HN, Vaillancourt B, Ou SJ, Jiang JM, Buell CR. Construction of a chromosome-scale long-read reference genome assembly for potato. GigaScience 2020, 9(9). 10.1093/gigascience/giaa100 PMC7509475 32964225

[41] Li H, Durbin R (2010). Fast and accurate long-read alignment with Burrows-Wheeler transform. Bioinformatics.

[42] Li H, Handsaker B, Wysoker A, Fennell T, Ruan J, Homer N, Marth G, Abecasis G, Durbin R (2009). Genome Project Data Processing S: the sequence Alignment/Map format and SAMtools. Bioinformatics.

[43] McKenna A, Hanna M, Banks E, Sivachenko A, Cibulskis K, Kernytsky A, Garimella K, Altshuler D, Gabriel S, Daly M (2010). The genome analysis Toolkit: a MapReduce framework for analyzing next-generation DNA sequencing data. Genome Res.

[44] Braz GT, He L, Zhao HN, Zhang T, Semrau K, Rouillard JM, Torres GA, Jiang JM (2018). Comparative Oligo-FISH mapping: an efficient and powerful methodology to reveal Karyotypic and chromosomal evolution. Genetics.

[45] Xiong Z, Gaeta RT, Edger PP, Cao Y, Zhao K, Zhang S, Pires JC. Chromosome inheritance and meiotic stability in allopolyploid Brassica napus. *G3* 2021, 11(2):jkaa011. 10.1093/g3journal/jkaa011 PMC8022990 33704431

